# Dual stack black blood carotid artery CMR at 3T: Application to wall thickness visualization

**DOI:** 10.1186/1532-429X-11-45

**Published:** 2009-11-10

**Authors:** Axel Bornstedt, Mathias Burgmaier, Vinzenz Hombach, Nikolaus Marx, Volker Rasche

**Affiliations:** 1 Department of Internal Medicine II - Cardiology, University Hospital Ulm, Albert-Einstein-Allee 23, Ulm, 89081, Germany

## Abstract

**Background:**

The increasing understanding of atherosclerosis as an important risk factor for the development of acute ischemic events like ischemic stroke has stimulated increasing interest in non-invasive assessment of the structure, composition and burden of plaque depositions in the carotid artery wall. Vessel wall imaging by means of cardiovascular magnetic resonance (CMR) is conventionally done by 2D dual inversion recovery (DIR) techniques, which often fail in covering large volumes of interest as required in plaque burden assessment. Although the technique has been extended to 2D multislice imaging, its straight extension to 3D protocols is still limited by the prolonged acquisition times and incomplete blood suppression. A novel approach for rapid overview imaging of large sections of the carotid artery wall at isotropic spatial resolutions is presented, which omits excitation of the epiglottis. By the interleaved acquisition of two 3D stacks with the proposed motion sensitized segmented steady-state black-blood gradient echo technique (MSDS) the coverage of the carotid artery trees on both sides in reasonable scan times is enabled.

**Results:**

10 patients were investigated with the proposed technique and compared to conventional transversal DIR turbo spin and gradient echo approaches centered at the height of the carotid bifurcation. In all MSDS experiments sufficient black-blood contrast could be obtained over the entire covered volumes. The contrast to noise ratio between vessel and suppressed blood was improved by 73% applying the motion sensitizing technique. In all patients the suspicious areas of vessel wall thickening could be clearly identified and validated by the conventional local imaging approach. The average assessable vessel wall segment length was evaluated to be 18 cm. While in 50% of the cases motion artifacts could be appreciated in the conventional images, none were detected for the MSDS technique.

**Conclusion:**

The proposed technique enables the time efficient coverage of large areas of the carotid arteries without compromising wall-lumen CNR to get an overview about detrimental alterations of the vessel wall. Thickening of the vessel wall can be identified and the suspicious segments can be targeted for subsequent high-resolution CMR. The exclusion of the epiglottis may further facilitate reduction of swallowing induced motion artifacts.

## Background

The well-established understanding of atherosclerosis as an important risk factor for the development of acute ischemic events has stimulated increasing interest in non-invasive assessment of the structure, composition and burden of plaque depositions in the carotid artery wall. Over the last decade, cardiovascular magnetic resonance (CMR) has proven to contribute substantially to the non-invasive assessment and characterization of atherosclerotic lesions, especially in the aorta and the carotid arteries [1-3].

For vessel wall imaging, black-blood contrast is required for ensuring sufficient contrast between the vessel wall and blood. Most commonly, suppression of the blood signal is achieved by applying a double-inversion recovery (DIR) black-blood preparation prepulse [4]. Most published approaches combine the DIR preparation with two-dimensional (2D) imaging by means of multi spin-echo (MSE) techniques [1-3]. Without compromising spatial resolution due to severe T_2 _apodization, this technique allows for the acquisition of a single slice in the minute time range, while providing sufficient signal to noise ratio (SNR) and lumen-wall contrast (CNR). Different image contrasts including T_2 _- (T2W), proton density (PDW) and, limited by the required inversion recovery time for blood signal nulling and heart rate, T_1 _- weighting (T1W) can be realized by slight modifications of the sequence timing. While the DIR technique has been extended to enable multislice acquisitions [5], its application for three-dimensional (3D) coverage of large sections of the arteries at sufficient isotropic spatial resolution has been limited. The resulting long acquisition times often cause image-distorting motion artifacts [6] due to swallowing, arterial pulsation and breathing. Incomplete blood suppression [7,8] caused by insufficient exchange of the blood in the entire volume may mimic incorrect wall thickening. Time-efficient 3D imaging of the carotid and aortic arteries has been achieved by applying segmented gradient echo techniques [9-11]. Swallowing motion artifacts have been addressed by utilization of navigators monitoring the position of the epiglottis [10,12] and arterial pulsation artifacts can be reduced by synchronizing the data acquisition with the heart beat. Improvement of blood suppression has been obtained by phase-sensitive reconstruction techniques [13] and diffusion-prepared or motion sensitized preparation techniques [10,9,14], all relying rather on the motion of the blood than on complete blood exchange between preparation and readout.

A major limitation of arterial wall imaging by CMR still results from the long acquisition times, which do not allow covering large sections of the arteries at sufficient spatial resolution for plaque characterization. Fast three-dimensional imaging methods, providing an overview on the arterial tree with sufficient spatial resolution and contrast, appear attractive for targeting the lesion of interest and planning of the subsequent high-resolution CMR plaque characterization protocol.

We recently developed a 3D localized volume-selective sequence for arterial wall imaging [15]. By combining a 2D pencil beam excitation with motion sensitized black-blood prepared imaging, large segments of the carotid artery tree could be covered in reasonable image acquisition times. Although the motion sensitizing prepulse causes a preweighting of the spins similar to a *T*_2 _preparation pulse of about 15 ms length, an excellent wall lumen contrast could be maintained and the reduced spatial resolution was still sufficient for assessment of the vessel wall thickness. Motion artifacts due to swallowing could not be observed, most-likely due to the local character of the excitation, which ensures that the region of the epiglottis does not contribute to the final MR image. Limitations of this technique rise from the rather long duration of the 2D excitation pulses, which is required to avoid substantial side-loops in the 2D excitation profile and the restriction to acquire a single carotid artery tree, only.

In this contribution, a new dual-stack approach is suggested, in which two parasagittally aligned 3D volumes (stacks) are acquired temporally interleaved. Black-blood contrast is achieved by motion-sensitized preparation, which is applied prior to the acquisition of each temporally interleaved stack. As in the local excitation approach, the proposed technique omits excitation of the epiglottis and is supposed to show similar advantages regarding swallowing artifacts. The efficiency of the motion-sensitizing magnetization preparation dual stack gradient echo (MSDS) sequence is demonstrated in patients for the rapid overview imaging of long segments of the carotid artery wall for both sides simultaneously. The applicability of the technique for identification of thickened vessel wall segments was investigated in direct comparison to the conventional high-spatial resolution protocols.

## Methods

The method was evaluated in 10 patients with a proven vascular disease defined as arteriosclerosis confirmed by the presence of coronary artery disease, peripheral artery disease or carotid plaque (10 males, mean age 58 +/- 7 y, mean weight 83 +/- 6 kg) without any contraindications for CMR. Written informed consent was obtained from all subjects before CMR and the protocol was approved by the ethics committee of the University.

All imaging was performed on a 3T whole-body system (Achieva, Philips Medical Systems, Netherlands) equipped with a high performance gradient system (40 mT m^-1^, 200 T m^-1 ^s^-1^). All data was acquired utilizing a dedicated two segment four-element carotid coil (Philips Research Europe, Germany). Either segment comprises two independent coil elements with spatial extent of 65 × 50 mm^2 ^each. The two segments were located on either side of the neck and fixated with a Vac-Lok neck cushion (Medtec, USA), which also immobilized the head of the patient to a great extent.

### Localizer

For localization, a multislice gradient-echo technique acquiring three orthogonal slabs with transversal, sagittal, and coronal slice orientation was applied. The resulting scout images were used for planning of the subsequent scans.

### Coil-Sensitivity Reference Scan

A coil-sensitivity reference scan was acquired, which was needed for the homogenization of the image signal and the vendor specific parallel imaging reconstruction, respectively.

### Angiograms

For targeting the position of the carotid arteries and bifurcations, fast thick slab phase contrast angiograms (see Table 1 for sequence details) were obtained in coronal and sagittal orientation.

**Table 1:** Scan parameters for the acquisitions performed.

	**PCA** **Survey**	**MSDS** **1.0 mm**	**MSDS** **0.8 mm**	**DIR-GRE**	**PD DIR-MSE**
**FOV mm (mps)**	300 × 300 × 50	150 × 200 × 30	150 × 200 × 30	150 × 150 × 20	150 × 150 × 20

**Resolution mm (mps)**	1.17 × 2.34 × 50	1.0 × 1.0 × 1.0	0.8 × 0.8 × 0.8	0.45 × 0.45 × 2.0	0.45 × 0.45 × 2.0

**Parallel Imaging Acc**.	NA	2.0 p	2.0 p/1.25 s	NA	2.0 p

**TE ms**	5.6	2.4	2.6	2.8	10

**TR/TR p. Segm. ms**	20/NA	5.2/1000	5.5/1000	5.4/1HB	NA/3 HB

**Flip angle**	15	20	20	20	90

**Turbo factor**	NA	40	40	30	12

**Signal Averages**	1	4	6	2	1

**Acq. Duration ms**	NA	206	222	161	125

**Scan duration s**	20	304	629	202	369

### Volume Imaging of the Carotid Arteries

**Figure 1:**
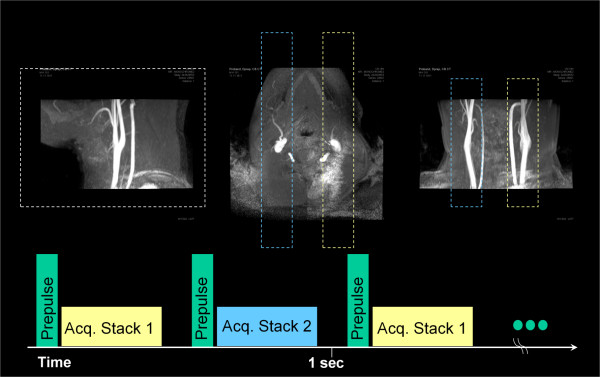
**Example of the location of the two stacks, planned on the thick-slab PCA images and acquisition order in MSDS**.

A 3D motion sensitized, fat suppressed, segmented, spoiled gradient echo dual stack sequence (MSDS) was developed. As reported previously [9,10,14,15], suppression of blood signal was accomplished by a motion sensitizing spin preparation. The preparation comprised a 90°_x_-180°_y_-90°_-x_-driven equilibrium technique with integrated balanced magnetic field gradients for dephasing the spins of moving blood. Suppression of residual transversal magnetization was achieved by subsequently applied spoiler gradients. The motion sensitizing or as previously called diffusion weighting strength (B-value) of the preparation pulse was chosen according to [10]. Overall preparation duration resulted as 21.5 ms for a cumulated B-value of 17.1 s mm^-2 ^in all three directions applying a 15 mT m^-1 ^gradient amplitude for 4.5 ms before and after the 180° refocusing pulse (slew rate 200 mT/(m ms), total gradient area 137 ms mT/m, first gradient moment 1029 mT ms^2^/m). The left and right carotid artery tree was covered by two independently planned sagittal volumes of dimension 150 × 30 × 200 mm at isotropic spatial resolution between 0.8^3 ^mm^3 ^and 1 mm^3^. Planning was done based on the phase contrast angiograms as shown in figure 1. During planning, it was ensured that the pharynx was not contained in the covered volumes of interest (VOI). To allow sufficient recovery of longitudinal magnetization, subsequent segments of the same stack were either acquired at a fixed user-defined spacing T_RS _of 700 - 1000 ms or a single RR interval. The acquisition of the second stack was interleaved and started T_RS_/2 after starting the acquisition of the first stack. The preparation was applied once prior to the acquisition of each segment (Figure 1 bottom) to ensure sufficient blood suppression in both stacks. Sequence details are provided in Table 1.

For comparison, a high-resolution proton-density weighted 3D double-inversion recovery multi spin echo sequence (DIR-MSE) and a 3D double-inversion recovery segmented gradient echo sequence (DIR-GRE) in axial view orientation were performed centered around the bifurcation of the respective carotid artery. For suppression of swallowing motion artifacts, a pencil beam navigator gating technique, initially developed for cardiac respiratory motion suppression [16], was applied in which the position of the epiglottis was monitored. Sequence details are provided in Table 1.

### Data Analysis

For image analysis, the data was transferred to a dedicated CMR image processing workstation (ViewForum, R2.6, Philips Medical Systems). The multi planar reformat tool was applied to define a path along the centerline of the common, external and internal carotid arteries (ACC, ACE and ACI). The endpoints of the paths were identified either at the location where the vessel exceeds the imaging volume or by a drop of the contrast C_W _between vessel wall and suppressed blood signal below 3 (). The length of the vessels was measured individually for the ACC (starting from the carina downwards) and the ACE/ACI (starting from the central ostia upwards).

Comparison of the suggested MSDS technique and the conventional DIR-MSE technique was done after axial reformatting of the MSDS data.

The effectiveness of the blood suppression of the motion sensitizing prepulse in comparison to the standard DIR technique was evaluated using the DIR-GRE and the MSDS data sets, as both sequences are based on nearly identical gradient echo acquisitions. Contrast to noise ratios, normalized to the voxelsize, were determined as follows: *CNR*_*norm *_= (*S*_*wall *_- *S*_*lumen*_)/(*stddev*_lumen _* *voxelsize*). The ROIs were drawn in the vicinity of the carotid bifurcation at corresponding locations to ensure that the coil sensitivity and intrinsic SNR properties were identical.

The performance of the new technique for the reduction of swallowing motion induced artifacts was qualitatively assessed by direct comparison of images acquired with the dual-stack approach with images acquired by the DIR-MSE and DIR-GRE acquisition techniques.

### Ultrasound Acquisition

Morphology of the ACC, ACI and ACE were displayed real-time with a high-resolution 9 MHz broadband linear array transducer (iU22 Ultrasound System, Philips Electronics, Bothell, WA, USA). Representative pictures were taken and compared to CMR.

## Results

**Figure 2:**
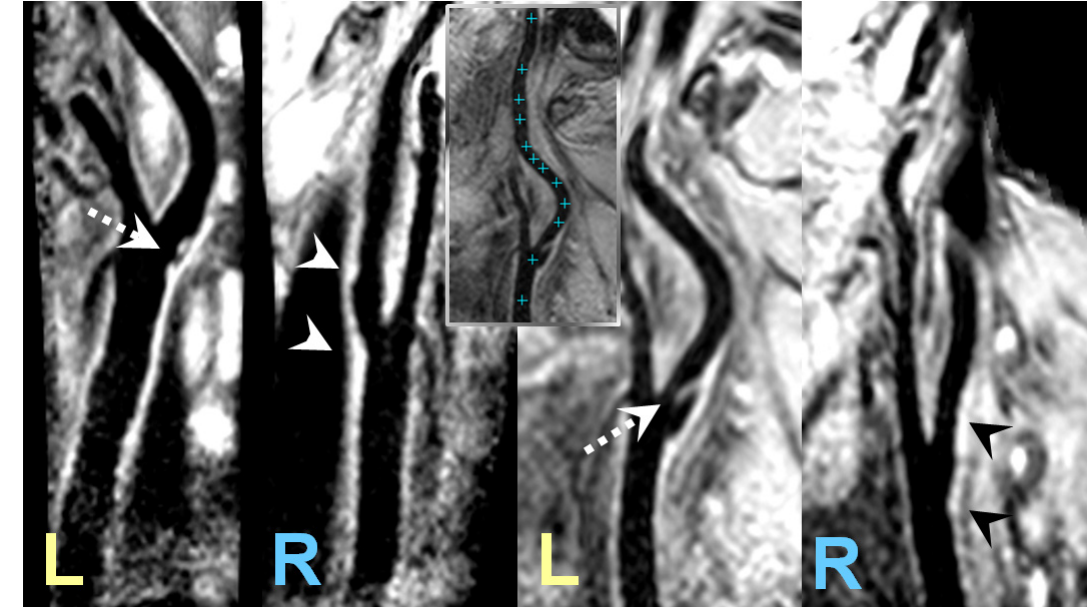
**Examples of dual stack (L: left side, R: right side) carotid imaging (reformats) acquired in two patients (left patient 0.8^3 ^mm^3^, right patient 1.0 mm^3 ^resolution)**. The images clearly reveal vessel wall thickening (arrow heads) as well as severe lesions (arrows). The inset demonstrates possible coverage and vessel length measurement on a curved MPR. The length of the indicated path (crosses) is 110 mm.

**Figure 3:**
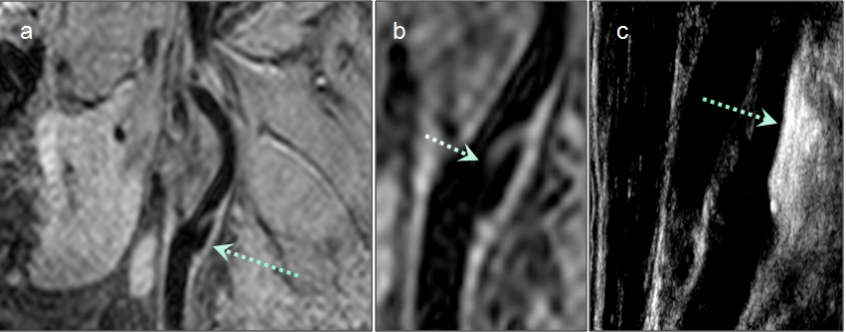
**Original CMR slice data cropped (a) and zoomed to lesion, indicated by arrows (b)**. The ultrasound image (9 MHz) at the according position, showing the severely calcified lesion in the left carotid artery bifurcation, is depicted in (c). Images correspond to the patient depicted on the right in Fig. 2.

The imaging experiments could be completed successfully in all subjects. In all experiments sufficient suppression of the blood signal could be obtained over the imaged VOI, while preserving the signal of the vessel wall. The *CNR*_*norm *_was 52 +/- 7 for the MSDS measurements, while it was 30 +/- 14 for the DIR-GRE; indicating the superior blood signal suppression and robustness of the motion sensitizing technique. Figure 2 shows two examples of reformatted images acquired at spatial resolutions of 0.8^3 ^and 1.0 mm^3^, demonstrating the obtainable image quality and coverage of the suggested technique. The arrows indicate atherosclerotic, partly calcified lesions, which were exemplarily confirmed by ultrasound. The inset demonstrates a vessel length measurement on a curved MPR indicating (crosses) a path of 110 mm length. Figure 3 shows a direct comparison of the original MSDS data to the ultrasound image at the according position, showing a severely calcified lesion in the left carotid artery bifurcation.

Visual inspection of the endovascular vessel wall revealed sufficient delineation over the entire accessible course of the carotid artery, while the contrast between the epivascular wall and the surrounding tissue was less pronounced, especially in the more distal sections of the ACE and ACI. No distinct motion artifacts (e. g. ghosting) introduced by swallowing could be observed.

The average length of the vessel wall, which could clearly be depicted, was 38.8 +/- 8.1 mm for the ACC, 77.5 +/- 14.6 mm for the ACI and 62.8 +/- 9.4 mm for the ACE. The overall assessable length (ACC + ACI + ACE) resulted to 179.1 +/- 18.1 mm for each of the 24 carotid vessel trees analyzed. The stopping criteria for the path length of the ACC was the drop of contrast and SNR respectively in 100% of the cases since the placement of the coil was restricted by the musculature of the neck. ACE/ACI paths could be followed to the border of the VOI in 83% of the cases.

**Figure 4:**
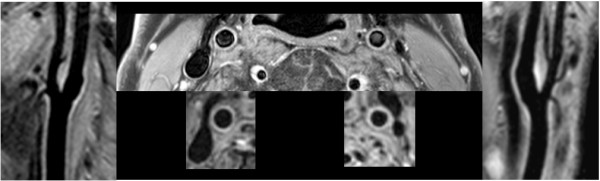
**Comparison of PD TSE DIR BB (top center) with motion sensitized dual stack (MSDS) GRE**. 0.45 mm vs. 0.8 mm in plane resolution.

In all patients vessel wall thickening or plaque could be seen in the DIR-MSE technique and could also be clearly identified from the MSDS images, indicating the applicability of the MSDS technique to fast identification of the location of atherosclerotic lesions. In one case, the full extent of the wall thickening was missed in the DIR-MSE images due to insufficient coverage of the distal section of the ACI. A direct comparison of DIR-MSE and MSDS images is provided in figure 4. Please note the superior blood suppression in the MSDS technique even so a substantially larger section of the carotid arteries was covered. Quantitative assessment of the vessel wall thickness in MSDS was limited by the achievable spatial resolution. While 1.0 mm^3 ^to 0.8^3^mm^3^, as reported here, appeared generally feasible, the acquisition of 0.7^3 ^mm^3 ^resolution datasets, tested in volunteers, yielded only satisfying results in subjects with vessels located in close proximity to the skin surface and thus the coil.

None of the MSDS images showed swallowing related image artifacts, where for the acquisition of the conventional double-inversion recovery multi-spin echo (DIR-MSE) and respective gradient-echo (DIR-GRE) techniques, navigators had to be used for avoiding severe motion artifacts caused by swallowing. Despite the navigator technique, in 50% of the DIR-MSE and DIR-GRE images motion artifacts were still observable in 50% of the cases.

## Discussion

In this work, a 3D motion-sensitized segmented dual-stack gradient echo (MSDS) technique was developed for fast black blood CMR of large sections of the carotid arteries. The motion-sensitized preparation revealed the potential for providing excellent blood signal suppression and contrast to noise ratios over large segments of the carotid arteries without obvious loss of signal intensity of stationary tissue and enabled dual stack acquisition without prolongation of the image acquisition time. Sufficient wall lumen contrast was achieved in the ACC, the ACE and the ACI enabling a clear delineation of the vessel wall over a mean length 179 +/- 18 mm. The interleaved acquisition of the right and left carotid artery tree by the suggested dual stack approach enabled rapid acquisition of an overview visualization of the carotid artery tree at isotropic spatial resolutions between 0.8^3 ^mm^3 ^and 1 mm^3 ^in less than 10 minutes. Compared to the alternative approach of acquiring a single coronal slab, the dual-stack approach is supposed to be less sensitive to motion artifacts caused by swallowing [6], since the region of the epiglottis is not included in the excited VOI and hence does not contribute to the final image. Cancellation of signal from blood by motion-sensitized preparation clearly holds the potential to generate black blood over large segments of the carotid arteries, which cannot be achieved by the competing DIR technique, since the blood is not totally exchanged over the large volumes addressed in this contribution. Even though, blood in the carotid arteries is mainly flowing in FH direction, best blood suppression was achieved by applying motion sensitizing gradients in all three spatial directions simultaneously.

The most promising application of the proposed MSDS technique is supposed to rise in the field of rapid global assessment of vessel properties and wall thickness in the carotid arteries for rapid identification of those locations along the vessel wall showing local thickening. Here, especially the isotropic spatial resolution, which enables almost lossless reformatting of the data along the centerline is supposed to be beneficial for identification of vessel wall thickening. Due to some limitations in spatial resolution, the proposed technique may not yet be suited to replace the well-established acquisition protocols for plaque characterization published earlier [1,3] and closer inspection of suspicious locations on the vessel wall with multi-contrast high resolution imaging is required for more detailed plaque analysis.

The application of MSDS for high-resolution imaging may become feasible with the advent of dedicated coils providing improved SNR in combination with better parallel imaging capabilities. Due to the coverage of large segments of the carotid arteries by the MSDS approach, patient motion between subsequent scans will not cause a targeted lesion to leave the VOI, as may be the case in conventional axially aligned DIR-MSE imaging approaches due to the limited coverage of the vessel in feet-head (FH) direction. This property of the MSDS technique may have further advantages with respect to longitudinal monitoring of plaque progression, since the larger covered VOI is supposed to ensure that the location of the lesion is covered completely even in case the VOI is not being planned perfectly the same. Furthermore, registrations based on a large VOI are supposed to be more precise than registrations performed on a small VOI. The suggested motion-sensitized dual-stack approach is not limited to gradient echo techniques and can be combined with other approaches for generating variable image contrast

## Conclusion

In conclusion, the 3D MSDS technique appears to be a very promising technique for rapid artifact-free overview imaging of the vessel wall of the carotid arteries with excellent black-blood contrast. Its application to high-resolution multi-contrast imaging is, in principle, possible but remains to be proven.

## Competing interests

The University Hospital Ulm and Philips Medical Systems have a collaborative research and development agreement for cardiovascular magnetic resonance imaging. No other financial conflicts of interest are identified.

## Authors' contributions

AB, VH, NM and VR have made substantial contributions to conception and design of the study and have been involved in drafting the manuscript or revising it. AB and VR have developed the methods and carried out the data analysis. MB was responsible for the ultrasound studies and revised the manuscript. All authors read and approved the final manuscript.

## References

[B1] Yuan C, Mitsumori LM, Beach KW, Maravilla KR (2001). Carotid atherosclerotic plaque: noninvasive MR characterization and identification of vulnerable lesions. Radiology.

[B2] Saam T, Hatsukami TS, Takaya N, Chu B, Underhill H, Kerwin WS, Cai J, Ferguson MS, Yuan C (2007). The vulnerable, or high-risk, atherosclerotic plaque: noninvasive MR imaging for characterization and assessment. Radiology.

[B3] Watanabe Y, Nagayama M, Suga T, Yoshida K, Yamagata S, Okumura A, Amoh Y, Nakashita S, Van Cauteren S, Dodo Y (2008). Characterization of atherosclerotic plaque of carotid arteries with histopathological correlation: vascular wall MR imaging vs. color Doppler ultrasonography (US). J Magn Reson Imaging.

[B4] Edelman RR, Chien D, Kim D (1991). Fast selective black blood MR imaging. Radiology.

[B5] Yarnykh VL, Yuan C (2003). Multislice double inversion-recovery black-blood imaging with simultaneous slice reinversion. Journal of Magnetic Resonance Imaging.

[B6] Boussel L, Herigault G, de la Vega A, Nonent M, Douek PC, Serfaty JM (2006). Swallowing, arterial pulsation, and breathing induce motion artifacts in carotid artery MRI. J Magn Reson Imaging.

[B7] Luk-Pat GT, Gold GE, Olcott EW, Hu BS, Nishimura DG (1999). High-resolution three-dimensional in vivo imaging of atherosclerotic plaque. Magn Reson Med.

[B8] Crowe LA, Gatehouse P, Yang GZ, Mohiaddin RH, Varghese A, Charrier C, Keegen J, Firmin DN (2003). Volume-selective 3D turbo spin echo imaging for vascular wall imaging and distensibility measurement. J Magn Reson Imaging.

[B9] Koktzoglou I, Li D (2007). Diffusion-prepared segmented steady-state free precession: Application to 3D black-blood cardiovascular magnetic resonance of the thoracic aorta and carotid artery walls. J Cardiovasc Magn Reson.

[B10] Koktzoglou I, Li D (2007). Submillimeter isotropic resolution carotid wall MRI with swallowing compensation: imaging results and semiautomated wall morphometry. J Magn Reson Imaging.

[B11] Lin H, Flask CA, Dale BM, Duerk JL (2007). Rapid dark-blood carotid vessel-wall imaging with random bipolar gradients in a radial SSFP acquisition. J Magn Reson Imaging.

[B12] Crowe LA, Keegan J, Gatehouse PD, Mohiaddin RH, Varghese A, Symmonds K, Cannell TM, Yang GZ, Firmin DN (2005). 3D volume-selective turbo spin echo for carotid artery wall imaging with navigator detection of swallowing. J Magn Reson Imaging.

[B13] Crowe LA, Varghese A, Mohiaddin RH, Yang GZ, Firmin DN (2006). Elimination of residual blood flow-related signal in 3D volume-selective TSE arterial wall imaging using velocity-sensitive phase reconstruction. J Magn Reson Imaging.

[B14] Wang J, Yarnykh VL, Hatsukami T, Chu B, Balu N, Yuan C (2007). Improved suppression of plaque-mimicking artifacts in black-blood carotid atherosclerosis imaging using a multislice motion-sensitized driven-equilibrium (MSDE) turbo spin-echo (TSE) sequence. Magn Reson Med.

[B15] Bornstedt A, Bernhardt P, Hombach V, Kamenz J, Spiess J, Subgang A, Rasche V (2008). Local excitation black blood imaging at 3T: application to the carotid artery wall. Magn Reson Med.

[B16] Wang Y, Rossman PJ, Grimm RC, Riederer SJ, Ehman RL (1996). Navigator-echo-based real-time respiratory gating and triggering for reduction of respiration effects in three-dimensional coronary MR angiography. Radiology.

